# Microplastics in freshwater ecosystems: what we know and what we need to know

**DOI:** 10.1186/s12302-014-0012-7

**Published:** 2014-07-09

**Authors:** Martin Wagner, Christian Scherer, Diana Alvarez-Muñoz, Nicole Brennholt, Xavier Bourrain, Sebastian Buchinger, Elke Fries, Cécile Grosbois, Jörg Klasmeier, Teresa Marti, Sara Rodriguez-Mozaz, Ralph Urbatzka, A Dick Vethaak, Margrethe Winther-Nielsen, Georg Reifferscheid

**Affiliations:** 1 Department of Aquatic Ecotoxicology, Goethe University Frankfurt am Main, Max-von-Laue-Str. 13, Frankfurt, 60438 Germany; 2 Catalan Institute for Water Research (ICRA), Girona, 17003 Spain; 3 Department Biochemistry and Ecotoxicology, Federal Institute of Hydrology, Koblenz, 56002 Germany; 4 Service Etat des Eaux Evaluation Ecologique, Agence de l’Eau Loire-Bretagne, Ploufragan, 22440 France; 5 Water, Environment and Eco-technologies Division, Bureau de Recherches Géologiques et Minières (BRGM), Orléans, 45100 France; 6 GéoHydrosystèmes Continentaux (GéHCO), Université Francois Rabelais de Tours, Tours, 37000 France; 7 Institute of Environmental Systems Research, Universität Osnabrück, Osnabrück, 49074 Germany; 8 Investigación y Proyectos Medio Ambiente S.L. (IPROMA), Castellón de la Plana, 12005 Spain; 9 Interdisciplinary Centre of Marine and Environmental Research (CIIMAR), Porto, 4050-123 Portugal; 10 Unit Marine and Coastal Systems, Deltares and Institute for Environmental Studies, VU University Amsterdam, Amsterdam, 1081 The Netherlands; 11 Environment and Toxicology, DHI, Hørsholm, 2970 Denmark

**Keywords:** Chemistry, Ecotoxicology, Environmental quality, Litter, Microplastics, Monitoring, Plastics, Polymers, Review, Water framework directive

## Abstract

**Background:**

While the use of plastic materials has generated huge societal benefits, the ‘plastic age’ comes with downsides: One issue of emerging concern is the accumulation of plastics in the aquatic environment. Here, so-called microplastics (MP), fragments smaller than 5 mm, are of special concern because they can be ingested throughout the food web more readily than larger particles. Focusing on freshwater MP, we briefly review the state of the science to identify gaps of knowledge and deduce research needs.

**State of the science:**

Environmental scientists started investigating marine (micro)plastics in the early 2000s. Today, a wealth of studies demonstrates that MP have ubiquitously permeated the marine ecosystem, including the polar regions and the deep sea. MP ingestion has been documented for an increasing number of marine species. However, to date, only few studies investigate their biological effects.

The majority of marine plastics are considered to originate from land-based sources, including surface waters. Although they may be important transport pathways of MP, data from freshwater ecosystems is scarce. So far, only few studies provide evidence for the presence of MP in rivers and lakes. Data on MP uptake by freshwater invertebrates and fish is very limited.

**Knowledge gaps:**

While the research on marine MP is more advanced, there are immense gaps of knowledge regarding freshwater MP. Data on their abundance is fragmentary for large and absent for small surface waters. Likewise, relevant sources and the environmental fate remain to be investigated. Data on the biological effects of MP in freshwater species is completely lacking. The accumulation of other freshwater contaminants on MP is of special interest because ingestion might increase the chemical exposure. Again, data is unavailable on this important issue.

**Conclusions:**

MP represent freshwater contaminants of emerging concern. However, to assess the environmental risk associated with MP, comprehensive data on their abundance, fate, sources, and biological effects in freshwater ecosystems are needed. Establishing such data critically depends on a collaborative effort by environmental scientists from diverse disciplines (chemistry, hydrology, ecotoxicology, etc.) and, unsurprisingly, on the allocation of sufficient public funding.

## Background

### Microplastics are freshwater contaminants of emerging concern

Among the multiple human pressures on aquatic ecosystems, the accumulation of plastic debris is one of the most obvious but least studied. While plastics generate remarkable societal benefits [1], there are downsides to our ‘plastic age’. Durability, unsustainable use, and inappropriate waste management cause an extensive accumulation of plastics in natural habitats [2]. In the marine environment, plastics of various size classes and origins are ubiquitous and affect numerous species that become entangled in or ingest plastics [3].

Under environmental conditions, larger plastic items degrade to so-called microplastics (MP), fragments typically smaller than 5 mm in diameter (see Table 1 for further information). Besides these degradation products (secondary MP), MP can also be produced as such (primary MP). For instance, MP are intentionally used as resin pellets (raw material for the production of plastic products) or as ingredient of personal care products (e.g., peelings and shower gels).

**Table 1:** **Classification of environmental (micro)plastics**

**Category**	**Description**
Classification	Environmental plastics are a *very heterogeneous* group of litter that can be characterized by various descriptors. In the literature, they are frequently stratified according to size, origin, shape, polymer type, and color. So far, there is no common classification system. Recently, the European MSFD Working Group on Good Environmental Status (WG-GES) provided a ‘Monitoring Guidance for Marine Litter in European Seas’ [76], which represents an important step towards a standardized sampling and monitoring of marine microplastics.
Size	The WG-GES defines size classes for plastic litter as follows: macroplastics (>25 mm), mesoplastics (5 to 25 mm), large microplastics (1 to 5 mm), and small microplastics (20 μm to 1 mm). Accordingly, items smaller than 20 μm will classify as nanoplastics.
Origin	Microplastics can also be categorized according to its origin: Primary microplastics are produced as such, for instance as resin pellets (raw materials for plastic products) or as additives for personal care products (e.g., shower gels and peelings). Secondary microplastics are degradation products of larger plastic items, which are broken down by UV radiation and physical abrasion to smaller fragments.
Polymers	The polymer type of environmental (micro)plastics can be determined by Fourier transformed infrared spectroscopy (FT-IR) or Raman spectroscopy. In concordance to global production rates, high- and low-density polyethylene (HD/LD-PE), polyethylene terephthalate (PET), polypropylene (PP), polystyrene (PS), and polyvinyl chloride (PVC) are the most common polymers found in the environment. In addition, polyamide fibers (nylon) from fishing gears are frequent.
Shape	The shape can be described according to the main categories: fragments (rounded, angular), pellets (cylinders, disks, spherules), filaments (fibers), and granules [76].

MP are of special concern since their bioaccumulation potential increases with decreasing size. MP may be ingested by various organisms ranging from plankton and fish to birds and even mammals, and accumulate throughout the aquatic food web [4]. In addition, plastics contain a multitude of chemical additives [5] and adsorb organic contaminants from the surrounding media [6]. Since these compounds can transfer to organisms upon ingestion, MP act as vectors for other organic pollutants [7] and are, therefore, a source of wildlife exposure to these chemicals [8,9].

Accordingly, MP are considered an emerging global issue by various experts [10,11] and international institutions [12,13]. These concerns and the public interest, however, focus almost exclusively on marine plastic debris. However, we argue that *microplastics are also freshwater contaminants of emerging concern*. This is supported by three arguments. First, although data is scarce, MP are present in freshwater ecosystems. Second, MP contain and adsorb micropollutants and pathogens. Third, laboratory studies demonstrate that marine organisms ingest MP and suffer adverse effect. While data on freshwater species is scarce, there is no reason to suppose that they remain unaffected. Thus, concerns about the impact of MP on freshwater ecosystems are legitimate and should receive more scientific attention.

### State of the science: focus on marine microplastics

So far, scientific efforts focus on marine MP, and studies on their abundance and effects become increasingly available. Because of its high mobility, plastic debris has practically permeated the global marine environment [14,15], including the polar regions [2], mid-ocean islands [16], and the deep sea [17]. Because of their specific hydrology, the large oceanic gyres are hot spots of plastic pollution (colloquially termed ‘garbage patches’), accumulating buoyant plastic debris. Here, the plastic abundance often exceeds that of zooplankton [18-21]. With respect to Europe's regional seas, MP have been reported for the Baltic, North, and Mediterranean Sea [22-25].

Most of the studies investigate neustonic and pelagic MP. However, MP are also present in sediments and have been detected on the shorelines and seafloors of six continents [15,26,27] with typical concentrations ranging from 1 to 100 items kg^−1^ [28]. A Belgian study reports a maximum of 400 items kg^−1^ in coastal harbor sediments [29]. Higher concentrations were reported in a Dutch study with 770 and 3,300 items kg^−1^ dry weight sediment in the Wadden Sea and the Rhine estuary, respectively [30]. Although abundant ubiquitously, the spatial distribution of MP in the marine environment is very heterogeneous [14]. This might be partly due to differences in methodology [28].

Field reports on detrimental interactions of plastics with biota (e.g., entanglement) are manifold [4]. However, only about a dozen studies have investigated MP uptake and effects under laboratory conditions, including two studies on freshwater species (literature search on ISI Web of Science, search term ‘microplastic*’ , manual filtering). With nine of these papers published since 2012, this is a very recent area of research. The ingestion of MP by marine invertebrates has been demonstrated in the laboratory for a broad spectrum of marine species: zooplankton [31-33], the lugworm *Arenicola marina* [34], the Blue mussel *Mytilus edulis* [35-37], and the sandhopper *Talitrus saltator* [38]. *M. edulis* is the only invertebrate in which the transfer of MP from the digestive tract to tissue has been studied and documented [35,36].

Data on the effects of MP exposure is limited. For zooplankton, a reduced algal feeding has been observed [31]. MP increased the mortality and decreased the fertility in copepods [32]. In the lugworm, MP reduced the weight and feeding and increased the bioaccumulation of plastic-associated polychlorinated biphenyls (PCBs) [34]. Reduced filtering activity and histological changes as response to inflammation have been reported for *M. edulis* [36,37], although another study did not find significant effects [35]. In the only study with marine vertebrates, the common goby *Pomatoschistus microps* was exposed to MP and pyrene [39]. MP delayed the pyrene-induced mortality but induced several toxicity biomarkers. In addition, two recent studies demonstrate the trophic transfer of MP along the marine food web from meso- to macrozooplankton [33] and from mussels to crabs [40].

## Discussion

### Presence of microplastics in freshwater ecosystems

Despite of the wealth of data on marine MP, to date, only a handful of studies investigate MP in a freshwater context. MP have been detected in the surface waters of the Laurentian Great Lakes [41]. The average abundance in the neuston was 43,000 items km^−2^, with a hotspot near metropolitan areas, which may represent important sources.

Three studies report the occurrence of MP in the sediments of lakes. Zbyszewski and Corcoran [42] found 0 to 34 plastic fragments m^−2^ on the shorelines of Lake Huron (Canada). Here, MP accumulation may be attributed to the lake's currents and nearby plastic manufacturers. Extending their shoreline monitoring to the Lakes Erie and St. Clair, Zbyszewski et al. [43] report 0.2 to 8 items m^−2^. Sampling two beaches of Lake Garda (Italy), Imhof et al. [44] found 100 and 1,100 MP items m^−2^ at the southern and northern shores, respectively. Similar to the Great Lakes, MP here consisted mainly of low-density polymers (polystyrene (PS), polyethylene (PE), and polypropylene (PP)).

Moore et al. [45] provide the first, non-peer-reviewed report on MP in rivers. In three Californian rivers, they found, on average, 30 to 109 items m^−3^. The midstream of the Los Angeles River carried 12,000 items m^−3^ and will discharge > 1 billion MP items day^−1^ into the Pacific Ocean. Although very limited, this data indicates that rivers transport relevant amounts of MP.

According to a recent study, the same is true for the second largest European rivers: Lechner et al. [46] used stationary driftnets and visual inspection to monitor plastic debris in the Austrian Danube. The authors report approximately 900 (2010) and 50 (2012) plastic items 1,000 m^−3^ in the size class of 0.5 to 50 mm. In a worst-case scenario, the Danube would discharge 4.2 t plastics day^−1^ and 1,500 t plastics year^−1^ to the Black Sea. The latter is more than the total plastic load of the whole North Atlantic Gyre [47]. Lechner et al. provide first evidence that large rivers transport significant amounts of (micro)plastics and thus contribute substantially to the marine plastics pollution.

**Figure 1:**
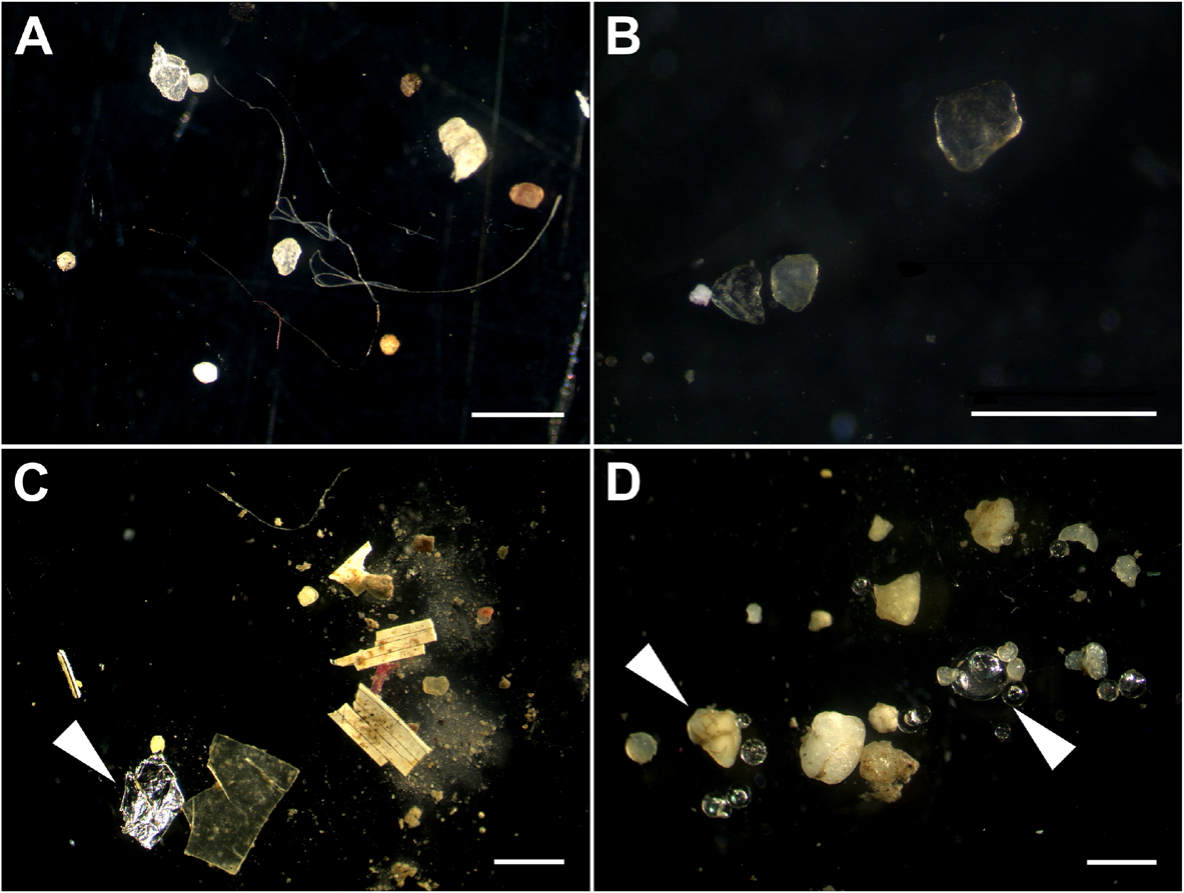
**Microplastics in sediments from the rivers Elbe (A), Mosel (B), Neckar (C), and Rhine (D).** Note the diverse shapes (filaments, fragments, and spheres) and that not all items are microplastics (e.g., aluminum foil **(C)** and glass spheres and sand **(D)**, white arrowheads). The white bars represent 1 mm.

Because data on the presence of MP in river sediments is lacking, the Federal Institute of Hydrology and the Goethe University carried out a small, exploratory study with sediments from the rivers Elbe, Mosel, Neckar, and Rhine (Germany). Using density separation and visual inspection, we found 34 to 64 MP items kg^−1^ dry weight, with the River Rhine containing the highest load. Plastic fragments accounted for 60% of the total MP; the remaining particles were synthetic fibers (Figure 1). Thus, as is the case for marine and estuarine sediments, river and lake sediments may be sinks for MP, deserving further investigation.

### Sources of microplastics

To date, the sources of marine MP are still not very well characterized. A rough estimation predicts that 70% to 80% of marine litter, most of it plastics, originate from inland sources and are emitted by rivers to the oceans [12]. Potential sources include wastewater treatment plants (WWTPs), beach litter, fishery, cargo shipping, and harbors [12,23,25,29,48]. Although data is so far unavailable, runoff from industrial plastic production sites may be an additional source. Taken together, most marine studies tentatively refer to inland waters as relevant sources (indeed they are rather transport pathways), while actual data is still scarce.

Inland sources of MP have not been investigated thoroughly. In analogy to the marine systems, major contributors will likely include WWTPs and runoff from urban, agricultural, touristic, and industrial areas, as well as shipping activities. Another potential source is sewage sludge that typically contains more MP than effluents [49]. Sewage sludge is still frequently used for landfilling and as fertilizer in agriculture, and surface runoff may transfer MP to rivers and lakes and ultimately river basins and the sea. Washing clothes [26] and personal care products [50] are sources of MP in WWTPs. Since the retention capacity of conventional wastewater treatment processes appears to be limited [14], a characterization of MP emission by WWTPs and other sources is urgently needed to understand where freshwater MP is coming from.

### Impact of microplastics on freshwater species

In a field report, Sanchez et al. [51] provide the only data on MP in freshwater fish so far. They investigated gudgeon (*Gobio gobio*) caught in 11 French streams and found MP in the digestive tract of 12% of the fish. Although again very preliminary, this field report shows that freshwater species ingest MP. However, the rate of MP ingestion in different fish species will certainly depend on their feeding strategy. Rosenkranz et al. [52] demonstrate that the water flea *Daphnia magna* rapidly ingests MP under laboratory conditions. MP (0.02 and 1 mm) appear to cross the gut epithelium and accumulate in lipid storage droplets. This is of specific concern because MP infiltrating tissues might induce more severe effects. Imhof et al. [44] report the uptake of MP by annelids (*Lumbriculus variegatus*), crustaceans (*D. magna* and *Gammarus pulex*), ostracods (*Notodromas monacha*), and gastropods (*Potamopyrgus antipodarum*). While the available studies demonstrate that a broad spectrum of aquatic taxa is prone to MP ingestion, the toxicological effects remain uninvestigated for freshwater species.

### Microplastics as vector for other contaminants

Due to their large surface-to-volume ratio and chemical composition, MP accumulate waterborne contaminants including metals [53] and persistent, bioaccumulative, and toxic compounds (PBTs) [54]. A review on the relationship between plastic debris and PBTs (e.g., PCBs and DDT) has been published recently [55], and a number of studies exist for polycyclic aromatic hydrocarbons (PAHs) [56-61]. However, there is a lack of information on other important contaminants like pharmaceuticals and endocrine-disrupting compounds (EDCs). Nonylphenol and bisphenol A have been detected in MP [60,62,63]. Fries et al. [24] detected various plastic additives in MP, including some well-known EDCs (e.g., phthalates). In addition, Wagner and Oehlmann [64,65] demonstrated that plastics leach EDCs. Since the spectrum of contaminants is different in freshwater and marine systems, the chemical burden of freshwater MP remains to be studied.

The interaction of MP and chemicals has been studied in adsorption-desorption experiments [6,57]. While there is significant complexity in this interaction, MP may act as vector transferring environmental contaminants from water to biota. While different modeling studies arrive at contrasting conclusions [54,66,67], a recent experimental study demonstrates that fish exposed to contaminants sorbed to MP bioaccumulate these compounds and suffer adverse effects (glycogen depletion and histopathological alterations [68]). However, to date, there are too few studies investigating whether MP are indeed vectors that facilitate the transfer of organic contaminants to biota. Because a verification of the ‘vector hypothesis’ would have major ecological implications, it deserves further investigation, especially in a freshwater context.

### Microplastics as vector for exotic species and pathogens

Not only the complex mix of chemicals contained in and sorbed to MP and/or ingestion of MP by biota is a cause for concern but also microorganisms developing biofilms on MP particles. Only very few studies have been conducted on this issue with marine ecosystems being the focal point of interest [69-72]. Zettler et al. [72] described a highly diverse microbial community (‘plastisphere’) attaching plastic marine debris in the North Atlantic. Several plastisphere members are hydrocarbon-degrading bacteria which may potentially influence plastic debris fragmentation and degradation. But they also found opportunistic (human) pathogens like specific members of the genus *Vibrio* dominating plastic particles. Therefore, MP can act as a vector for waterborne (human) pathogens influencing the hygienic water quality. The fact that the microbial communities on MP are distinct from surrounding water (only some marine bacteria develop biofilms on microplastic particles (e.g., [71,72])) suggests that MP serve as a kind of new habitat. Until now, the complex interaction between microorganisms/microbial communities as a key player in aquatic ecosystems/food webs and MP, especially in freshwater, is poorly understood and needs to be further investigated.

### Microplastics in connection to European water policies

The issue of (micro)plastics connects to several European water policies. The European Marine Strategy Framework Directive (MSFD, 2008/56/EC) addresses the issue of marine litter, including plastics. Here, MP are covered by Descriptor 10 of Commission Decision 2010/477/EU, which defines the good environmental status of marine waters [73].

In contrast, the Water Framework Directive (WFD, 20/60/EC) applying to European inland waters does not specifically refer to plastic litter. However, the Member States have the obligation to monitor anthropogenic pressures. Here, MP are promising candidates, especially because they might act as vectors for a wide range of freshwater contaminants. For instance, MP have been shown to contain the WFD priority substances di(ethylhexyl) phthalate (DEHP), nonylphenol, octylphenol, and PAHs (2008/105/EC, Annex II).

Several other European Directives relate to the potential sources of freshwater MP, including the Directives on packaging waste (2004/12/EC), waste (2008/98/EC), landfills (1999/31/EC), urban wastewater (91/271/EEC), sewage sludge (86/278/EEC), and ship-source pollution (2005/35/EC). In addition, the Union's chemicals legislation (REACH, 1907/2006/EC) will apply to plastic monomers and additives of relevant production volumes.

In a recent ‘Green paper on a European strategy on plastic waste in the environment’, the European Commission addresses the issue as part of a wider review of its waste legislation [74]. While the Green Paper focuses on potential mitigation strategies for plastic litter at the source, it also expresses ‘particular concern’ about MP.

## Conclusions

### Knowledge gaps and research needs

The investigation of (micro)plastics in aquatic environments is a highly dynamic and interdisciplinary area of research covering and bringing together the disciplines of oceanography and hydrology as well as environmental monitoring, modeling, chemistry, and toxicology. In recent years, this collaborative effort advanced our understanding of the environmental impact of MP, especially by providing extensive monitoring data. Ongoing research activities focus, however, almost exclusively on marine MP.

**Figure 2:**
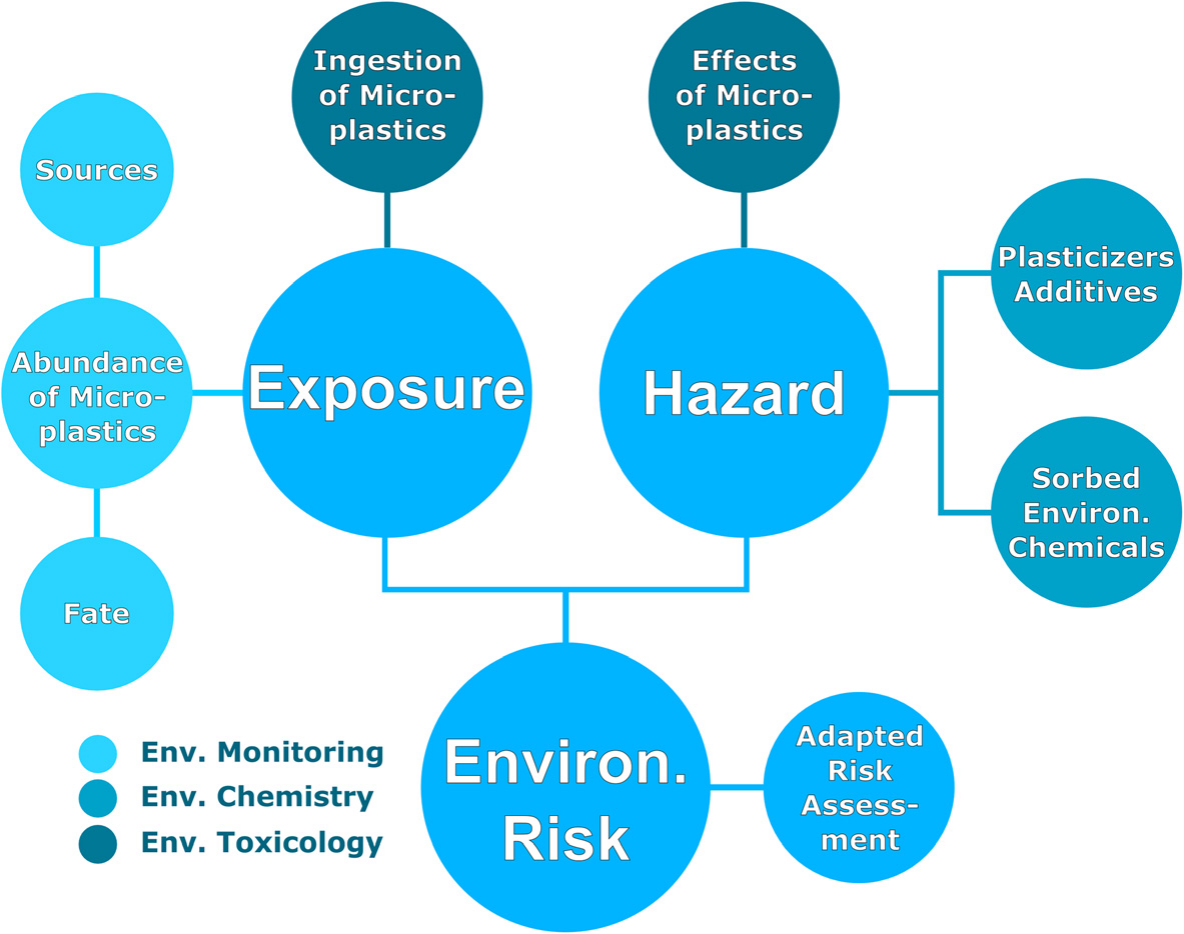
**Research aspects with regard to freshwater microplastics.** All areas need to be investigated more thoroughly to assess the environmental risk associated with microplastics in freshwater ecosystems.

Data on freshwater ecosystems is at best fragmentary if not absent. This lack of knowledge hampers a science-based environmental risk assessment of freshwater MP. Such assessment is needed to facilitate a societal and political discussion at national and European levels on the issue, which, depending on the outcome, will result in mitigation measures eventually. For instance, MP could be integrated as descriptor of environmental status in the WFD. However, environmental scientists first need to close the gaps of knowledge with regard to exposure and hazard of freshwater MP and the associated chemicals. Based on the current state of the science, the following research needs emerge (Figure 2):

1. Monitoring the presence of microplastics in freshwater systems. While few studies on large lakes and rivers are available, we have no clear picture on the magnitude of the plastics pollution in surface waters. Generating comprehensive monitoring data on the abundance of freshwater MP is needed to understand their environmental impact.
2. Investigating the sources and fate of freshwater microplastics. Currently, we still do not understand the behavior of MP in aquatic ecosystems. Based on data on their abundance, modeling approaches are needed to identify hotspots and sinks and quantify loads. One important aspect of understanding the environmental fate is also to identify relevant inland sources of MP and determine the fragmentation rates of large plastic debris.
3. Assessing the exposure to microplastics. With evidence coming from marine species, it appears plausible that freshwater organisms will ingest MP, too. However, actual data is scarce. Environmental toxicologists need to determine the intake of MP by freshwater key species. It will be crucial to understand which plastic characteristics (size, material, and shape) promote an uptake and what is the fate of MP in the biota (e.g., excretion, accumulation, and infiltration of tissues). These aspects need to be studied under laboratory conditions and in the field to determine the actual exposure.
4. Evaluating the biological effects of microplastics exposure. Besides abundance and exposure, the question whether MP induce adverse effects in organisms is crucial to determine their environmental hazard. In the absence of effect studies on freshwater species, one can only speculate on potential sensitive endpoints: Ingested plastic fragments may most likely affect the metabolism (starvation due to decreased energy intake) and induce inflammation (when transferring to tissues). Because this is an area of research where the least progress has been made so far, the investigation of MP effects on marine and freshwater species need to be intensified considerably.
5. Understanding the interaction between microplastics and other freshwater contaminants. Plastics itself can contain and release toxic chemicals (e.g., monomers or plastic additives [75]). In addition, they can accumulate environmental chemicals from the surrounding. This may increase the chemical exposure of the ingesting organism and, thus, toxicity. The findings on chemicals associated with marine MP (mostly POPs) cannot be transferred to freshwaters because here the spectrum and concentrations of pollutants is very different. Therefore, it is important to investigate the chemical burden of freshwater MP, including the absorption/desorption kinetics and the transfer of chemicals from plastics to biota.
6. Develop a novel framework for the risk assessment of microplastics. MP can be direct and indirect stressors for the aquatic environment: They are contaminants of emerging concern *per se* and, in addition, may serve as vectors for invasive species and for other pollutants. To account for that, the classical risk assessment framework needs to be adapted. For instance, the mixture toxicity of MP-associated compounds and the modulation of the compounds' bioavailability need to be integrated.

There are some challenges in investigating these aspects: To generate commensurable data on the abundance of freshwater MP, harmonized monitoring procedures, including sampling, identification, and characterization, are needed. For that, the ‘Monitoring Guidance for Marine Litter in European Seas’ developed by the European MSFD Working Group on Good Environmental Status [76] provides an excellent starting point. The separation of MP from the sample materials (sediments or suspended particulate matter) and the confirmation of the plastics' identity to avoid misclassification is still a very resource-consuming and biased process (e.g., when visually identifying MP in complex samples). Here, sample throughput and accuracy need to be increased. Likewise, we need to improve the capability to detect very small MP in the low micrometer range. Boosting technological innovation in the area of MP research (e.g., coupling of microscopy and spectroscopy to identify very small MP) will help meet those challenges.

In conclusion, based on our knowledge on the environmental impact of marine MP, their freshwater counterparts should be considered contaminants of emerging concern. However, there is a considerable lack of knowledge on MP in surface waters worldwide. Data on their presence, sources, and fate is scarce if not absent. The same is true for their chemical burden and biological effects. To enable science-based environmental risk assessment of freshwater MP, it is imperative to initiate coordinated and collaborative research programs that close these gaps of knowledge.

## Abbreviations

EDCs: endocrine-disrupting compounds
FT-IR: Fourier transformed Infrared spectroscopy
HD/LD-PE: high-/low-density polyethylene
MP: microplastics
MSFD: European Marine Strategy Framework Directive
PAHs: polycyclic aromatic hydrocarbons
PBTs: persistent, bioaccumulative, and toxic compounds
PCBs: perchlorinated biphenyls
PET: polyethylene terephthalate
POPs: persistent organic pollutants
PP: polypropylene
PS: polystyrene
PVC: polyvinyl chloride
WFD: European Water Framework Directive
WWTPs: wastewater treatment plants
